# Optimisation of Polyphenols Extraction from Wild Bilberry Leaves—Antimicrobial Properties and Stability Studies

**DOI:** 10.3390/molecules28155795

**Published:** 2023-08-01

**Authors:** Ana-Maria Brezoiu, Mihaela Deaconu, Raul-Augustin Mitran, Ana-Maria Prelipcean, Cristian Matei, Daniela Berger

**Affiliations:** 1 Faculty of Chemical Engineering and Biotechnologies, University Politehnica of Bucharest, 1-7 Gheorghe Polizu Street, 011061 Bucharest, Romania; ana_maria.brezoiu@upb.ro (A.-M.B.); mihaela.deaconu@upb.ro (M.D.); cristian.matei@upb.ro (C.M.); 2 “Ilie Murgulescu” Institute of Physical Chemistry, Romanian Academy, 202 Splaiul Independentei, 060021 Bucharest, Romania; raul.mitran@gmail.com; 3 National Institute of R&D for Biological Sciences, 296 Splaiul Independetei, 060031 Bucharest, Romania; anamaria.prelipcean@incdsb.ro

**Keywords:** design of experiments, bilberry, polyphenolic extract, chemical stability, antimicrobial properties

## Abstract

Polyphenolic extracts from natural sources have received great interest due to their beneficial properties for human health. A method to reduce their variability is to use the design of experiments which allows a limited number of experiments to be performed while exploring the experimental space. Firstly, a 2^3^-full factorial model was used to investigate the polyphenols extraction from wild bilberry leaves. Spectrophotometric data (the content of polyphenols, flavonoids, chlorophyll and radical scavenger activity) and extraction yield were used as responses, and six statistical models were determined depending on the two numerical factors (temperature and alcohol % of ethanol–water mixture) being significant (*p* < 0.05) in all cases. Numerical optimisation performed by Design Expert 13 software correlates well with the chemical profile determined by high-performance liquid chromatography and the amount of the polyphenol. Afterwards, under the optimised conditions, an extract was prepared in three extraction steps for which composition, chemical stability and antimicrobial properties were evaluated. The antimicrobial potential of the extract was compared with that of the standard compounds (rutin and chlorogenic acid), and the results supported a synergistic effect of the extract components.

## 1. Introduction

There is a general trend of people improving their diet by using foods with high amounts of beneficial compounds. Bilberries are well known for their health benefits among fruits [1]. Thus, a three- to four-fold increase in their production was observed in Romania between 2015 and 2020 [2], as well as in other European countries [3]. Bilberry fruits [4], like other forest fruits (blackberries, raspberries, strawberries, mulberries, black chokeberries [5], etc.), are highly studied for their antioxidant, anti-inflammatory and antimicrobial properties, as well as for antidiabetic activity [6,7].

The consumption of fresh fruits is the typical way of achieving their positive impacts on human health. However, controversy is arising over the consumption of fresh fruits or their valorisation in extracts. It is up for debate whether extracts exhibit better effects to justify the additional processing efforts [8].

Several residues from bilberry cultivation or processing are available, such as pomace from the juice factories, twigs and leaves. Efforts are therefore being made to develop products from these residues with high levels of phytocompounds (mainly catechins, proanthocyanidins, phenolic acids, flavonols, etc.) [9], while allowing waste to be disposed of in a safe manner without causing pollution usually associated with waste incineration [10].

The variability associated with cultivation conditions, climate and processing parameters are important concerns in the development of products from natural compounds. The design of experiments allows for a limited number of experiments to be performed in order to investigate the experimental space and determine the optimal extraction parameters. Such approaches can reduce the impact of the variability associated with the development of natural compound products.

There are a limited number of studies focused on the design of phytocompounds extraction, mainly on non-edible residues, such as sea buckthorn leaves [11,12,13], winery by-products [14,15], apples [16] and potato peels [17,18,19]. The polyphenols extraction from bilberry pomace was studied through central composite design and response surface methodology (RSM) [20], while bilberry fruit extraction using the Box–Behnken design [7] or RSM [21] was also reported. A study of bilberry leaf extract after LED irradiation was also performed [22].

The preservation or improvement of the properties of natural products should be achieved during their development. In addition, the stability over time of their components should be investigated [23].

Herein we report a good correlation between the chemical profile of polyphenolic extracts prepared from wild bilberry twigs and leaves, determined by spectrophotometric and HPLC characterisation, and by modelling experiments using factorial design. The optimisation of the extraction process was then performed. The optimum conditions were then used for the preparation of another extract. These conditions included three extraction steps for maximum yield of the desired compounds. The chemical stability was investigated, while the evaluation of the antimicrobial properties of the extract showed a synergistic effect between the main components.

## 2. Results and Discussion

### 2.1. Design of Experiments

A factorial experiment was considered since the extraction parameters influence the properties of the extracts. The investigated factors include a temperature range of 30–60 °C, corresponding to low or medium energy consumption, nontoxic solvents (ethanol, or 1/1 (*v*/*v)* ethanol–water mixture) and conventional (Conv.) or ultrasound-assisted extraction (US). These factors and their levels are shown in Table 1, while the eight coded experiments (2^3^ full factorial designs) are listed in Table 2. Eight 1 h extraction experiments using a vegetal material/solvent ratio of 1/10 (*w*/*v*) were performed. The solids were filtered off under a vacuum, and the extracts were dried to constant mass using a rotary evaporator (RE100-Pro, DLAB Scientific, Beijing, China) after the extraction time had elapsed. The solids were further redissolved using the same solvent, and then the polyphenolic extracts were analysed to determine their chemical profile.

### 2.2. Spectrophotometric Characterisation of the Extracts

The total polyphenols, flavonoids and chlorophyll content values were determined through UV-Vis spectroscopy methods. The DPPH and ABTS methods were used to assess the antioxidant capacity of the prepared extracts. Extracts prepared by conventional extraction at 30 °C in ethanol or ethanol–water mixture were considered as references for statistical analysis in all spectrophotometric determinations.

**Figure 1:**
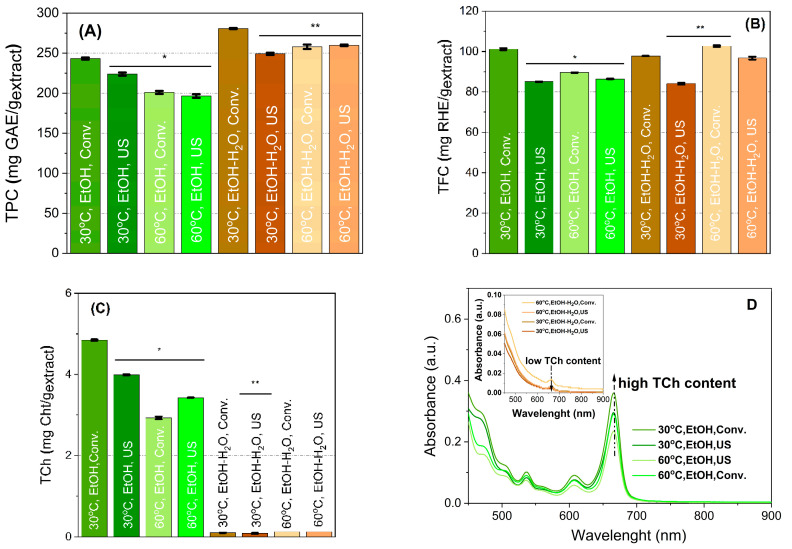

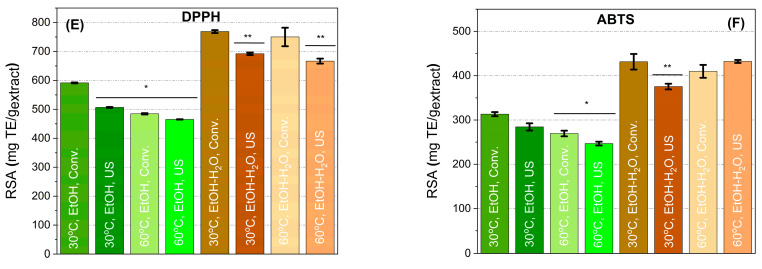
Spectrophotometric evaluation of bilberry extracts. Total polyphenols content (**A**), total flavonoid content (**B**), total chlorophyll content (**C**), UV-Vis spectra of extracts for chlorophyll determination (**D**), antioxidant activity using DPPH (**E**) and ABTS assay (**F**) (significantly different compared to conventional extraction at 30 °C performed in either ethanol (*) or ethanol–water mixture (**)—Student *t* test, *p* < 0.05).

The total polyphenol content (TPC) was expressed in gallic acid equivalents (GAE)/g extract (Figure 1A). The solvent type greatly influences the number of polyphenols, being higher in the case of the ethanol–water mixture than for ethanol. This fact can be explained by the increased polyphenols solubility because of the water addition [24]. The TPC values (Figure 1A) range from 196.48 ± 2.27 mg gallic acid equivalents (GAE)/g extract (178.53 ± 2.07 mg caffeic acid equivalents (CAE)/g extract) to 280.69 ± 0.73 mg GAE/g extract (255.08 ± 0.67 mg CAE/g extract).

The highest number of polyphenols was obtained for the conventional extraction at 30 °C in 1/1 ethanol/water. Increasing the temperature led to a decrease in the total polyphenols content in the case of ethanol extraction assisted by ultrasound. A contribution of the temperature and the applied extraction method could also be noticed, as all samples are significantly different from the reference. The obtained TPC values are higher than those previously reported by Stefanescu et al. for hydroethanolic extracts prepared by ultrasound-assisted extraction (UAE) from different species of bilberry leaves (89.40–155.67 mg GAE/g) [25], by Routray et al. for microwave hydroethanolic extracts in acidified solvent with or without LED irradiation (138.90–160.96 mg GAE/g) [22] or for UAE in acidified methanol from 73 different species of bilberry leaves (47.16–211.60 mg GAE/g) [26].

The total flavonoid content (TFC) (Figure 1B), expressed in rutin hydrate equivalents (RHE)/g extract, ranges from 6.661 to 31.285 mg RHE/g plant (2.208 to 10.367 mg of QE/g plant). These values are lower than those reported by Stefanescu et al. (31.36–67.88 mg QE/g of plant) [25]. Li et al. also reported lower TFC content values for fruit extracts (0.162–0.512 mg of RHE/g extract), with higher TFC values obtained for leaf extracts than for fruits [27].

A significant difference between the extracts prepared in the two solvents is related to their total amount of chlorophyll (TCh) (Figure 1C). Chlorophyll was better recovered in absolute ethanol, at low temperature, using the conventional method (2.93–4.85 mg CHt/g) than in 1/1 ethanol/water mixture, where it was barely present (0.09–0.13 mg CHt/g) (Figure 1D). The UV-Vis spectra of the extracts showed the absence of chlorophyll bands (665 and 649 nm) when the ethanol–water mixture was used as extraction solvent (Figure 1D inset). Nonetheless, the best TCh values are higher than those reported for 80% acetonic extracts prepared from bilberry leaves grown on fertilised soils (1.18–1.75 mg/g) [28].

Polyphenolic extracts were characterised for their ability to interact with free radicals using DPPH and ABTS assays (Figure 1D,F). The radical scavenger activity expressed as Trolox equivalents (TE)/g varied between 465.16 and 591.44 mg TE/g (DPPH), and 246.10 and 313.20 mg TE/g (ABTS) for ethanolic extracts. Higher values were obtained for hydroethanolic extracts (666.67–768.58 mg TE/g and 375.34–432.11 mg TE/g for DDPH and ABTS, respectively), in agreement with their TPC values. The antioxidant activity of the prepared extracts is similar to that reported by Wu et al. for a large number of leaf extracts from different bilberry species (143.60–586.60 μmol TE/g–DPPH and 89.00–754.83 μmol TE/g—ABTS) [26].

### 2.3. Experiments Modelling and Analysis of Variance (ANOVA)

An analysis of variance was performed for each response. The equations describing the extraction yield, TPC, TFC, TCh, as well as RSA (DPPH and ABTS assays) were determined. The equations (Table 3) show which factors, or first-order interactions cause a positive effect on the yield (positive numbers), and which reduce it. The equations containing the temperature (A), solvent concentration (B) and extraction method (C) parameters are listed in Table 3.

**Figure 2:**
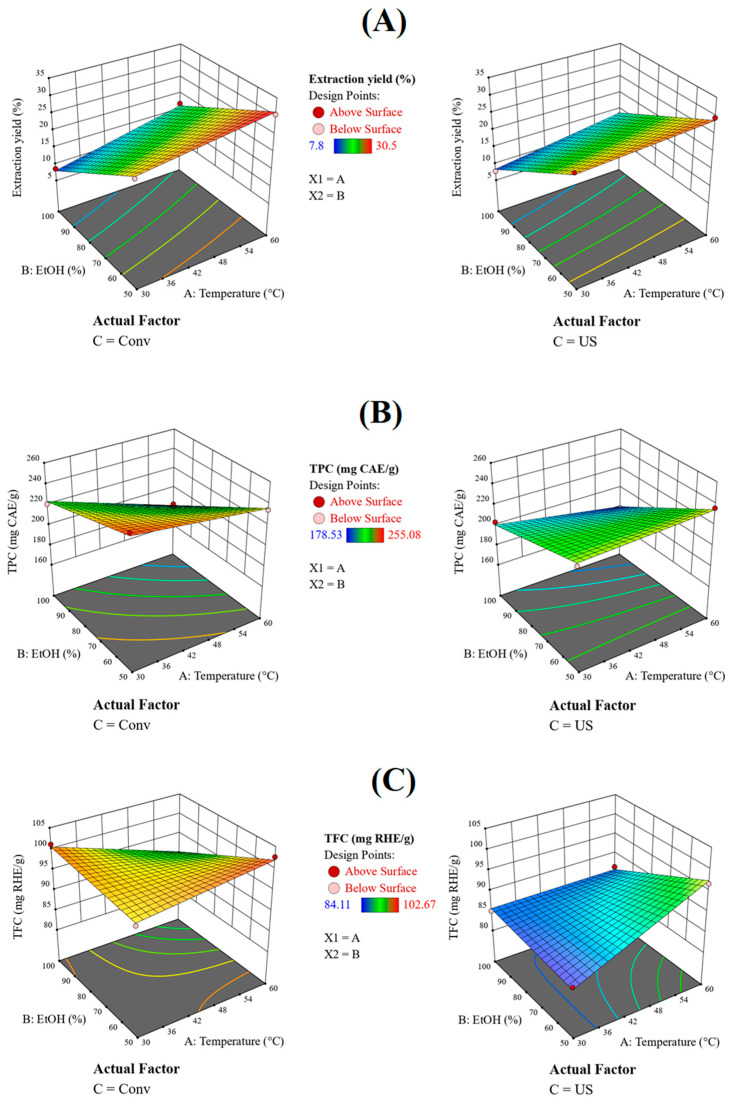

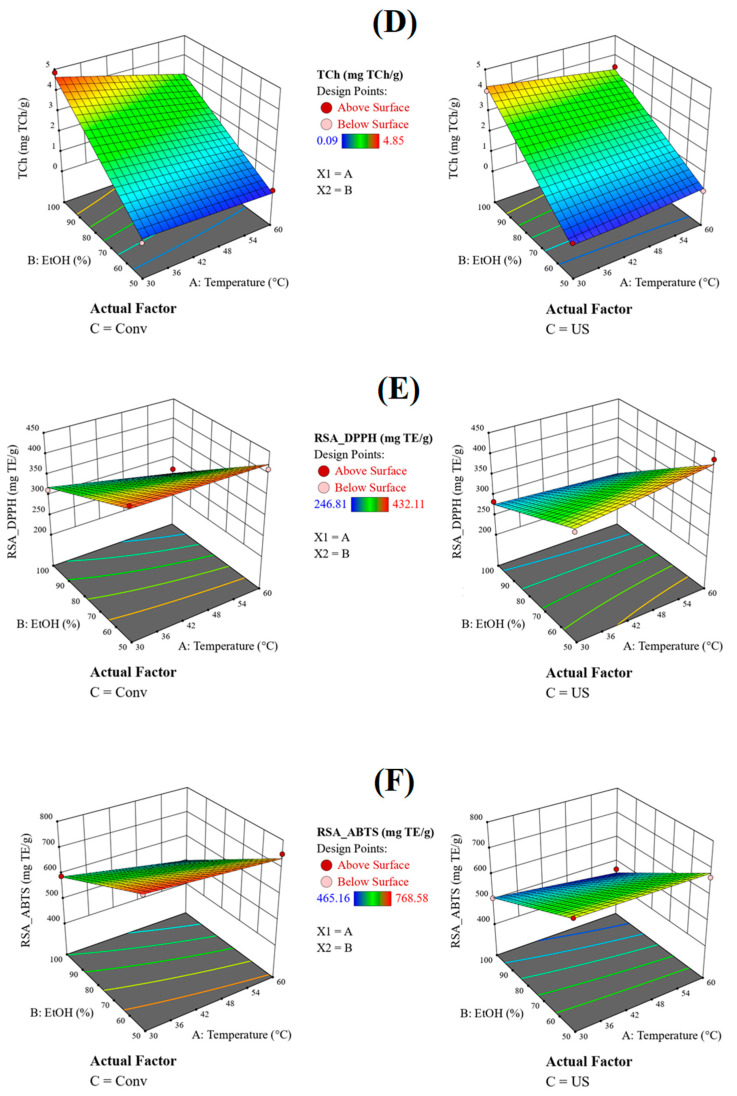
Three-dimensional surface plots showing the response dependence on numerical factors for (**A**)—extract yield, (**B**)—total polyphenols content, (**C**)—total flavonoid content, (**D**)—total chlorophyll content, (**E**)—antioxidant activity (DPPH method) and (**F**)—antioxidant activity (ABTS assay) (Red and blue colours represent high and low levels of phytocompounds or higher and lower antioxidant activity, respectively).

It can be noticed that the extraction method influences the extraction yield (Table 3), with a contribution from the extraction temperature (A). The process yield has higher values when the extraction temperature increases in 1/1 EtOH/H_2_O regardless of the extraction method (Figure 2A).

Increasing the extraction temperature usually decreases the amount of the total polyphenol, except in the case of the hydroethanolic solvent extraction assisted by US treatment (Figure 2B).

The TFC values show a different trend in ethanolic extracts than for hydroethanolic extracts. A decrease in TFC values with increasing temperature can be noticed for the ethanolic extracts, while the reverse holds true for hydroethanolic extracts (Table 3, Figure 2C).

The total chlorophyll amount is mostly dependent on the solvent (Table 3), showing the same slope for both conventional and ultrasound-assisted extraction. A TCh decrease with increasing temperature was noticed for ethanol, while the TCh values increased for the hydroethanolic mixture (Figure 2D).

The radical scavenging activity of the extracts determined by the DPPH assay is in agreement with the TPC trend previously discussed. An increase in antioxidant activity can be noticed for low temperatures in ethanol through the ABTS method (Figure 2E). The inverse dependence was seen for the hydroalcoholic mixture (Table 3, Figure 2F).

Good correlation coefficients were obtained in all cases (Table 3). These equations can therefore be used to predict the characteristics of extracts prepared in absolute or 50% ethanol mixtures, in the 30–60 °C temperature range, through conventional or ultrasound-assisted extraction.

### 2.4. Numerical Optimisation

Several studies have assumed that the optimal extraction conditions depend on the group of phytocompounds of interest [29,30]. Numerical optimisation was performed using the desirability function. This objective function takes values from zero to one. Performing numerical optimisation in Design Expert involves finding the run (extraction conditions) that maximises the desirability function. Hence, a numerical optimisation revealed the best extraction conditions if certain responses are maximised. The optimal parameters are presented in Table 4. The identified runs (or prepared extracts) exhibited high desirability values (0.962–1.000). It was then investigated which extraction parameters lead to the highest desirability in maximising all responses, assuming that each response has equal importance. The best polyphenols extraction conditions were achieved by conventional extraction at 60 °C in 50% ethanol, resulting in a good desirability value of 0.784.

### 2.5. Extracts Composition Determined by HPLC–PDA

The chemical profile of polyphenolic extracts was determined by HPLC–PDA, which allowed the identification of up to five phytocompounds: catechin hydrate, (-) epicatechin, rutin hydrate from flavonoid group, chlorogenic acid from hydroxycinnamic acid class in ethanolic extracts and caffeic acid in hydroethanolic extracts. The presence of caffeic acid could also be explained by the hydrolysis of chlorogenic acid (Table 5).

The ethanolic extracts (Runs 1–4) had high levels of catechin hydrate (4.156–5.517 mg/g), chlorogenic acid (61.894–75.207 mg/g), epicatechin (14.110–15.498 mg/g) and lower amount of rutin hydrate (14.814–16.372 mg/g) than that of hydroethanolic extracts (Runs 5–8) (32.271–46.650 mg/g). Caffeic acid was detected only in the hydroalcoholic extracts (0.575–1.882 mg/g). The highest level of polyphenols was noticed for Run 7 (conventional extraction at 60 °C in ethanol–water solvent) in agreement with the numerical optimisation. These amounts are comparable and mostly higher than those reported by Stefanescu for 40% hydroethanolic extracts prepared by ultrasound-assisted extraction from five varieties of bilberry leaves from Romania. The chemical profile of the obtained extracts encompasses catechin hydrate (5.21–7.27mg/g), chlorogenic acid (0.44–1.23 mg/g), epicatechin (up to 4.19 mg/g), caffeic acid (2.62–5.93 mg/g) and rutin hydrate (14.814–35.77 mg/g) [25].

### 2.6. Evaluation of the Extract Chemical Stability

An extract under optimised extraction conditions (Run 7, 60 °C, EtOH 50%, conventional extraction) was further prepared in three extraction steps with solvent replacement (extract denoted E7×3). Three extraction steps were performed to ensure that the vegetal material was depleted of the phytocompounds of interest. The extract had a high level of polyphenols with an extraction yield of 42.8% wt. (267.99 ± 4.3 mg CAE/g extract), a flavonoid content of 130.95 ± 1.16 mg RHE/g extract and a total chlorophyll amount of 0.34 ± 0.01 mg Cht/g extract. The antioxidant activity of the E7×3 extract has higher values compared to the optimised run from the factorial model, 696.76 ± 5.89 mg TE/g extract (DPPH assay) and 441.47 ± 18.25 mg TE/g extract (ABTS method). The chemical profile of extract determined by HPLC–PDA contains chlorogenic acid (87.463 ± 0.331 mg/g), caffeic acid (1.211 ± 0.003 mg/g) and rutin hydrate (64.103 ± 0.211 mg/g). Catechin hydrate and epicatechin were not identified in the E7×3 extract in comparison with the E7 extract, probably due to their lower chemical stability [31], while a higher amount of rutin hydrate was quantified.

The chemical stability of the E7×3 extract was assessed using HPLC and TG-DTA analyses. The evaluation of the E7×3 extract stability was performed by treating the sample at 40 °C and 75% relative humidity (RHu) for up to 28 days. The extract was characterised by thermogravimetric analysis after 7, 14, and 28 days of treatment and compared with the untreated E7×3 extract. All samples showed similar thermal behaviour (Table 6). When the extract was heated from 25 °C to 150 °C, a mass loss accompanied by an endothermic event was noticed, which is characteristic of water or residual solvent evaporation (Supplementary Information, Figure S1). Two superimposed mass loss effects can be noticed in the 150–300 °C temperature range, with no significant heat change. These superimposed mass losses can be assigned to the decomposition of volatile components of the extract. A pronounced exothermic event attributed to the combustion of organic substances can be seen in the 300°–650 °C temperature range. The samples have 1–3% wt. dried residue at 1000 °C, explained by the formation of inorganic components.

**Figure 3:**
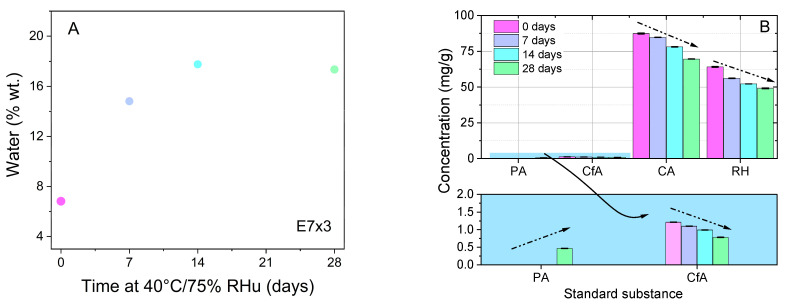
Stability of E7×3 extract at 40 °C, 75% RHu- (**A**) Water content determined by TG-DTA and (**B**) results of HPLC–PDA analysis (PA—protocatechuic acid, CfA—caffeic acid, CA—chlorogenic acid, RH—rutin hydrate).

The water content of the E7×3 extract, determined by TG-DTA analysis (Table 6), from the first event of the DTA curve (Figure 3A), was used to determine extract concentration prior to its dissolution for HPLC–PDA analysis (Figure 3B). The chemical profile determined after 7, 14 and 28 days of treatment showed a decrease in chlorogenic acid (from 87.463 to 69.591 mg/g extract), caffeic acid (from 1.211 to 0.781 mg/g extract) acid and rutin hydrate (from 64.103 to 49.056 mg/g extract) amounts, while protocatechuic acid (initially not detected) was identified in the extract after 28 days (0.468 ± 0.003 mg/g). The formation of protocatechuic acid is probably explained by the hydrolysis of a polyphenol with a higher molecular weight (Supplementary Information, Figure S2).

The antioxidant activity assessment after 10 months of storage of E7×3 samples showed that the extract stored in dark conditions at 4 °C better preserved the radical scavenging properties (with a loss of 12.6%—DPPH and 14.2%—ABTS of initial RSA value), compared to samples initially exposed to high humidity at 40 °C for 7, 14 or 28 days and then stored at 4 °C. The latter suffered a greater loss of antioxidant activity (52.5–54.9%—DPPH and 45.9–56.1%—ABTS of initial RSA value; Supplementary Information, Figure S3). The antioxidant activity is consistent with the chemical profile of extracts, with a larger number of phenolic compounds being associated with a higher RSA value.

### 2.7. Biological Evaluation of the E7×3 Extract

The antimicrobial properties of the E7×3 extract were tested in comparison with the main standard substances present in the extract in the same concentration as in the extract. All tested samples showed concentration-dependent antimicrobial activity. The assay addressed both the planktonic and biofilm growth states and used culture-based approaches.

#### 2.7.1. Antibacterial Activity on Planktonic Growth

**Figure 4:**
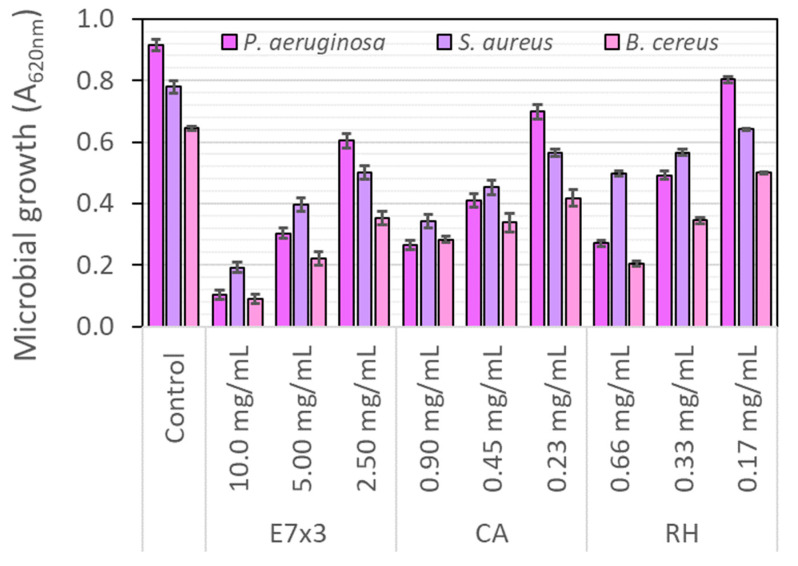
Effect of bilberry extract (E7×3) and standard compounds (CA—chlorogenic acid, RH—rutin hydrate) on planktonic bacterial growth after 24 h of treatment. All samples were statistically different from Control, as well as the extract compared to the standard compounds in the same amount (Student *t*-test, *p* < 0.05).

The data obtained on bacterial planktonic growth indicate an efficient inhibition at the highest concentration tested, with the bacterial growth inhibition having a strong dependence on concentration. The trend was maintained for all three bacterial strains. The most susceptible bacterial strain proved to be *P. aeruginosa*, a Gram-negative strain. Hence, the potential mechanisms of the antibacterial activity of bilberry extract could be the permeabilisation of the bacterial membrane or the inhibition of the efflux pump activity. The interesting finding of this study is the synergy between chlorogenic acid and rutin hydrate present in the extract (Figure 4). Inhibition of microbial growth was considerably higher in the case of bilberry extract when compared to the individual components, chlorogenic acid and rutin hydrate. There are studies that reported antibacterial components in bilberry extracts against periodontopathic bacteria, *Porphyromonas gingivalis*, *Fusobacterium nucleatum* and *Prevotella intermedia* [32].

#### 2.7.2. The Antibacterial Activity on Biofilm Formation

**Figure 5:**
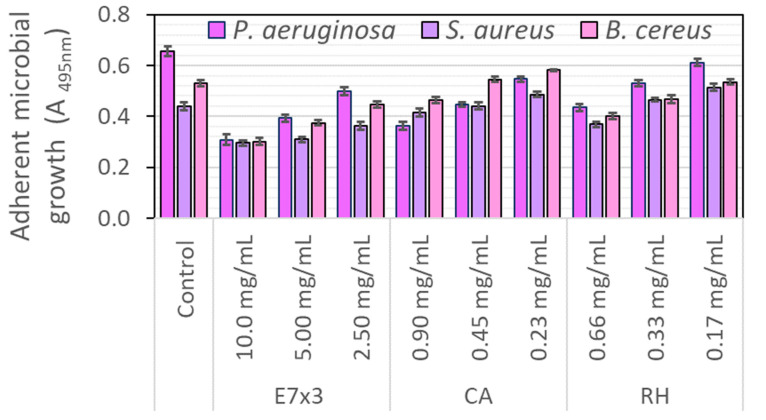
Effect of bilberry extract (E7×3) and standard compounds (CA—chlorogenic acid, RH—rutin hydrate) on bacterial biofilm formation and growth after 72 h of treatment. All samples were statistically different from Control, as well as the extract compared to the standard compounds in the same amount (Student *t*-test, *p* < 0.05).

In terms of adherent microbial growth, the tested bacteria seemed more resistant; only the highest concentration tested had the potential to interfere with bacterial biofilm formation and development. It is interesting to note that *P. aeruginosa* susceptibility was also maintained in terms of adherent growth, with significant suppression of the bacterial biofilm being detected at 72 h (Figure 5). Similar behaviour was observed for *B. cereus*, a Gram-positive strain involved in foodborne pathogenesis.

On the other hand, *S. aureus*, which is a commensal and opportunistic pathogen causing mild to life-threatening infections from superficial skin infections to invasive diseases in both humans and animals [33], showed a more robust resistance even for 10 mg/mL of the bilberry extract. Biofilm formation is associated with several virulence factors and increased tolerance against diverse antibiotics and antibacterial agents.

The synergistic effect observed for the bilberry extract containing both chlorogenic acid and rutin was detected in the case of adherent growth too. The synergistic effects are highly important given the goal of new pharmaceutical formulations in using the most effective compounds with lower concentrations and the development of the best combination. The synergistic effect of chlorogenic acid and caffeic acid with fosfomycin (a novel and promising antibiotic) on growth inhibition of resistant *Listeria monocytogenes* strain was reported [34].

## 3. Materials and Methods

### 3.1. Materials

The vegetal material used to obtain the extracts were *Vaccinium myrtillus* twigs and leaves, harvested in August 2021 from the spontaneous wild flora of the Cindrel Mountains (Batrana peak) at 1850 m altitude. The materials were air-dried to constant mass, considering the high levels of polyphenols and flavonoids with good antioxidant activity [26,27].

Sodium carbonate (Na_2_CO_3_), Folin–Ciocalteu reagent, 2,2-diphenyl-1-picrylhydrazyl (DPPH), potassium persulphate (K_2_S_2_O_8_) and 2,2’-azino-bis (3 ethylbenzothiazoline-6-sulphonic acid) (ABTS) purchased from Sigma–Aldrich (Sigma–Aldrich Co. Merck Group, Darmstadt, Germany) and 6 hydroxy-2,5,7,8-tetramethylchroman-2-carboxylic acid (Trolox, 97%, Aldrich Chemical Co Inc., Milwaukee, WI, USA) were used as received without further purification in the spectrophotometric determinations.

Standard HPLC-grade substances from phenolic and hydroxycinnamic acids, flavonoids, tannins, stilbenes, as well as anthocyanidins groups, were used for chromatographic analyses: caffeic acid (98%, HPLC, Sigma, Merck Group, Darmstadt, Germany), caftaric acid (Molekula GmbH, Munich, Germany), catechin hydrate (>98%, HPLC, Sigma, Merck Group, Darmstadt, Germany), chlorogenic acid (primary reference standard, HWI group, Alpen Aan de Rijn, The Netherlands), chicoric acid (>98%) from TCI (Tokyo, Japan), cyanidin chloride (>95%, HPLC, Sigma, Merck Group, Darmstadt, Germany), delphinidin chloride (analytical standard, Sigma–Aldrich Co. Merck Group, Darmstadt, Germany), gallic acid (98%, Alfa Aesar, Ward Hill, MA, USA), (−) epicatechin (>98%, HPLC, TCI, Tokyo, Japan), ellagic acid dihydrate (>98%, HPLC, TCI, Tokyo, Japan), gallic acid (98%, Alfa Aesar, Ward Hill, MA, USA), kaempferol (>97%, HPLC) from Sigma (Merck Group, Darmstadt, Germany), malvidin chloride (>95%, HPLC, Sigma–Aldrich Co. Merck Group, Darmstadt, Germany), myricetin (>96%, HPLC-grade), pelargonidin chloride (Aldrich Chemical Co Inc., Milwaukee, WI, USA), protocatechuic acid (>98%, HPLC, TCI, Tokyo, Japan), quercetin (>95%, HPLC), rosmarinic acid (>98%, HPLC, Sigma, Merck Group, Darmstadt, Germany), rutin hydrate (95%, HPLC), syringic acid (>98.5%, Molekula GmbH, Munich, Germany), trans-p-coumaric acid (analytical standard, Sigma–Aldrich Co. Merck Group, Darmstadt, Germany), trans-ferulic acid (>98%, GC) and trans-resveratrol (certified reference material, Sigma–Aldrich Co. Merck Group, Darmstadt, Germany). Solvents such as ethanol, acetonitrile (ACN) (Riedel-de Haën, Honeywell Riedel-de Haën, Seelze, Germany) and formic acid (Merck Group, Darmstadt, Germany) were used as received. Ultrapure water (Millipore Direct-Q3 UV water purification system with Biopack UF cartridge) was used for all aqueous solutions and experiments.

### 3.2. Methods

Conventional (Conv.) and ultrasound-assisted extraction (US; Bandelin Sonorex Digitec ultrasonic bath—Berlin, Germany) were applied to obtain the ethanolic and hydroalcoholic extracts.

The spectrophotometric and HPLC–PDA analyses were carried out as previously reported [35,36,37]. For the total phenolic content (TP) determination, a standard curve for gallic acid was established based on the absorbances at two wavelengths, 650 nm (y = 0.00965x + 0.013; R^2^ = 0.9998) and 765 nm (y = 0.00945x + 0.017; R^2^ = 0.9995) and the values were presented as an average of four determinations. The total flavonoid content (TF) was evaluated by plotting a standard curve for rutin hydrate at 410 nm (y = 0.0135x, R^2^ = 0.9990). For the determination of chlorophyll a (Cha) and b (Chb) content, three samples of each extract with the same concentration were used. The UV-Vis spectrum of each sample was recorded, and the solution absorbance values at 665, 649, and 750 nm were used in Ritchie’s equations to determine the total amount of chlorophyll (Cht).

Cha = [13.5275 · (A_665nm_ − A_750nm_) − 5.201 · (A_649nm_ − A_750nm_) · f · S] ·C^−1^ (1)

Chb = [22.4327 · (A_649nm_ − A_750nm_) − 7.0741 · (A_665nm_ − A_750nm_) · f ·S] ·C^−1^ (2)

Cht = Cha + Chb(3)

where Cha—chlorophyll a in mg/g, Chb—chlorophyll b in mg/g, Cht—the total amount of chlorophyll in mg/g, A—absorbance of the solution at a certain wavelength, f—dilution factor and S—the volume of solvent (ethanol or ethanol–water = 1/1 *v*/*v*) and C—concentration of the extract (mg/mL) [38].

The radical scavenger activity (RSA) evaluation was performed using a standard curve of Trolox established in mg of Trolox equivalent (TE)/mL extract, based on the absorbances at 517 nm (y = 178.12x + 1.69; R^2^ = 0.9983)—DPPH method and at 753 nm (y = 325.55x + 2.29; R^2^ = 0.9980)—ABTS assay, and the results were presented as an average of three replicates [35].

The chemical profile of prepared extracts was assessed by reverse phase high-performance liquid chromatography (HPLC; Shimadzu Nexera 2, Shimadzu Corporation) with a photodiode array detector (SPD-M30A, Shimadzu Corporation) in the wavelength range of 250–600 nm, using a Nucleoshell^®^ reversed-phase C18 column (Macherey-Nagel GmbH & Co. KG, Düren, Germany) with 4.6 mm × 100 mm (2.7 µm), a gradient elution at constant flow of 0.4 mL/min at 20 °C and using an injection volume of 1 µL. In total, 2.5% (*v*/*v*) aqueous formic acid water solution (A) and 90% (*v*/*v*) aqueous acetonitrile with 2.5% formic acid (B) were used as the mobile phases: the details regarding the chromatographic analyses, elution program and the standard substances elution in the applied method were described elsewhere [35,36,37].

The design of experiments and modelling was carried out in Design Expert 13 software using a factorial model of 2^3^ with one replicate and one block in which two numerical factors, temperature and ethanol concentration, and a categorical one, extraction method, were used. Two levels were considered for every factor, and all experimental spectrophotometric determinations, as well as the extraction yield, were chosen as model responses. The modelling was performed using ANOVA, and the model was considered significant for *p* < 0.05. Statistical analysis of the spectrophotometric data was conducted using Student’s *t*-test on each pair of interest. Differences were considered statistically significant for *p* < 0.05.

The stability of the bilberry leaves extract was evaluated using a controlled humidity atmosphere (75% relative humidity—RHu) created in a desiccator using a saturated NaCl solution (1 part salt/2 parts water), and the samples weighed in open Eppendorf containers were placed on top of the Petri dish with the saturated NaCl solution and incubated at 40 °C for 7, 14 and 28 days. The temperature and time were chosen in agreement with the World Health Organization’s recommendation for stability studies in temperate climates [39]. They were subsequently analysed by thermogravimetric analysis coupled with differential thermal analysis (TG-DTA, GA/SDTA851e from Mettler Toledo, Greifensee, Switzerland) and their moisture increase over time was evaluated.

Antibacterial activity on planktonic growth. The antibacterial activity of the extracts was evaluated on both Gram-positive, *Staphylococcus aureus* (ATCC 25923), *Bacillus cereus* (ATCC 11778) and Gram-negative, *Pseudomonas aeruginosa* strains (ATCC 27853). Samples were sterile-filtered. *S. aureus* and *B. cereus* were grown on trypticase soy agar (TSA) nutrient medium, while *P. aeruginosa* was grown on Luria Bertani agar at 37 °C. The overnight culture was diluted to a final concentration of 1 × 10^8^ colony-forming units per mL (CFU/mL) in each well containing 10, 5 and 2.5 mg/mL of bilberry extracts and the corresponding rutin and chlorogenic acid. After 24 h, the absorbance of the supernatant was assessed at 620 nm to determine the bacterial viability using a Sunrise microplate reader (Tecan).

Antibacterial activity on biofilm formation. The antibacterial potential of the bilberry extracts against biofilm formation was evaluated spectrophotometrically by measuring the number of bacterial cells adhering to the scaffolds. Briefly, bacterial suspensions were seeded at a density of 1 × 10^8^ CFU/mL in each well in a 96-well plate. The number of bacteria from the 3 strains attached to the plastic wells was measured spectrophotometrically. After 72 h, each well was washed 3 times in sterile phosphate-buffered saline (PBS; pH 7.2) to remove planktonic cells, while the bacteria attached to the substrate were fixed with methanol, stained with aqueous crystal violet 1% solution and decoloured with acetic acid 33%. The optical density of each well stained with crystal violet was measured at 495 nm using a Sunrise plate reader (Tecan).

## 4. Conclusions

Extracts with high levels of polyphenols, flavonoids and chlorophyll exhibiting good antioxidant activity were obtained, and the variation in their composition was studied using a Student *t*-test on each pair of interests. A 2^3^-factorial model was developed using Design Expert software, which had all spectrophotometric data as responses, as well as the extract yield. A good correlation was achieved between experimental and predicted values in all cases (R^2^ ranging from 0.9827 to 0.9948). In addition, numerical optimisation was carried out, and the conventional extraction in 1:1 (*v*/*v*) ethanol–water mixture at 60 °C had the highest desirability in maximising all model responses.

The extract with the best properties determined by the model was found to have the highest number of polyphenols (flavonoids and hydroxycinnamic acid derivatives), which emphasises a good correlation between the numerical optimisation and the chemical profile of the extracts.

An extract E7×3 was prepared in three extraction steps based on the optimisation results. It showed improved antimicrobial properties, especially against the *P. aeruginosa* strain, than that of the main individual standard compounds of the extract, while the chemical stability studies proved that storage of the extract at high humidity alters its chemical composition with an important reduction in the number of phytocompounds.

A potential application for food or nutraceuticals is possible when taking into account the high levels of antioxidants and good antimicrobial activity of the prepared extracts.

## Figures and Tables

**Table 1:** Design of experiments matrix.

Factor	Factor Code	Coded Level	Level
Temperature	A	−1	30 °C
		+1	60 °C
Solvent	B	−1	EtOH 100%
		+1	EtOH 50%
Extraction method	C	−1	Conv.
		+1	US

**Table 2:** Full factorial design experiments.

Run	A	B	C
1	−1	−1	−1
2	−1	−1	+1
3	−1	+1	−1
4	−1	+1	+1
5	+1	−1	−1
6	+1	−1	+1
7	+1	+1	−1
8	+1	+1	+1

**Table 3:** Analysis of variance for response prediction using factorial design.

Extraction yield (%)=19.99+2.51⋅A−8.39⋅B−0.5125⋅C−0.8875⋅AB−0.6875⋅AC (*R*^2^ = 0.9948, *p* = 0.0130)	
***Conventional extraction***	***Extraction yield*** (%) = 44.05 + 0.035833·***A*** *−* 0.442·***B*** + 0.002367·***AB***
***Ultrasound extraction***	***Extraction yield*** (%) = 47.15 − 0.055833·***A*** − 0.442·***B*** + 0.002367·***AB***
TPC (mgCAE/g)=217.20−9.31⋅A−20.79⋅B−6.10⋅C+6.54⋅AB+5.48⋅AC (*R*^2^ = 0.9924, *p* = 0.0188)	
***Conventional extraction***	***TPC* (mgCAE/g)** = 271.15750 + 0.322167·***A*** − 0.0469·***B*** − 0.017443·***AB***
***Ultrasound extraction***	***TPC* (mgCAE/g)** = 226.10250 + 1.05217·***A*** − 0.0469·***B*** − 0.017443·***AB***
TFC (mg RHE/g)=92.93+0.9112⋅A−2.38⋅B−4.86⋅C+3.46⋅AB+2.58⋅AC (*R*^2^·=·0.9912, *p* = 0.0218)	
***Conventional extraction***	***TFC* (mgRHE/g)** = 78.815 − 0.58075·***A*** + 0.32·***B*** − 0.00923 ***AB***
***Ultrasound extraction***	***TFC* (mgRHE/g)** = 53.58 + 0.92525·***A*** + 0.32 ***B*** − 0.00923 ***AB***
TCh (mgCht/g)=1.95−0.305⋅A+1.85⋅B−0.05⋅C+0.315⋅AB+0.1675⋅AC (*R*^2^ = 0.9915, *p* = 0.0212)	
***Conventional extraction***	***TCh* (mgCht/g)** = −4.9575 + 0.0315·***A*** + 0.1117·***B*** − 0.00084·***AB***
***Ultrasound extraction***	***TCh* (mgCht/g)** = −6.0625 + 0.05383·***A*** + 0.1117·***B*** − 0.00084·***AB***
$RSA_{DPPH}\;(\mathrm{mgTE/g})=345.33-5.76\cdot A-66.83\cdot B-10.66\cdot C+14.55\cdot AB+10.56\cdot AC$ (*R*^2^ = 0.9827, *p* = 0.0426)	
***Conventional extraction***	***RSA_DPPH_* (mg/TE/g)** = 474.5125 + 1.82183·***A*** − 0.9277·***B*** − 0.038793·***AB***
***Ultrasound extraction***	***RSA_DPPH_* (mg/TE/g)** = 389.8575 + 3.22917·***A*** − 0.9277·***B*** − 0.038793·***AB***
$RSA_{ABTS}\;(\mathrm{mg/TE/g})=615.62-24.02\cdot A-103.67\cdot B-33.03\cdot C+13.08\cdot AB$ (*R*^2^ = 0.9898, *p* = 0.0252)	
***Conventional extraction***	***RSA_ABTS_* (mg/TE/g)** = 936.08 + 0.52833·***A*** − 2.5778·***B*** − 0.034867·***AB***
***Ultrasound extraction***	***RSA_ABTS_* (mg/TE/g)** = 825.96 + 1.50317·***A*** − 2.5778·***B*** − 0.034867·***AB***

Factors: A—temperature, B—EtOH (%), C—extraction method. *p* < 0.05 means that the models are significant.

**Table 4:** Numerical optimisation using Design Expert software.

Optimal Extraction Conditions	Temperature (°C)	Solvent	Extraction Method	Desirability
Extraction yield (%)	60	EtOH–H_2_O	Conv.	1.000
TPC	30	EtOH–H_2_O	Conv.	0.964
TFC	60	EtOH–H_2_O	Conv.	0.962
TCh	30	EtOH	Conv	0.998
RSA_ABTS_	60	EtOH–H_2_O	Conv.	1.000
RSA_DPPH_	30	EtOH–H_2_O	Conv.	1.000
All responses	60	EtOH–H_2_O	Conv.	0.784

**Table 5:** HPLC–PDA composition of bilberry extracts.

	Catechin Hydrate	Chlorogenic Acid	Caffeic Acid	(-) Epicatechin	Rutin Hydrate
RT (min)	12.751 ± 0.009	13.469 ± 0.011	15.521 ± 0.006	17.686 ± 0.011	27.435 ± 0.012
Run 1	4.462 ± 0.044	75.207 ± 0.222	nd	14.851 ± 0.094	16.372 ± 0.016
Run 2	4.156 ± 0.025	61.849 ± 0.233	nd	14.110 ± 0.098	14.739 ± 0.083
Run 3	4.918 ± 0.004	65.866 ± 0.457	nd	14.824 ± 0.095	15.454 ± 0.104
Run 4	5.517 ± 0.022	74.039 ± 0.017	nd	15.498 ± 0.008	14.814 ± 0.007
Run 5	4.456 ± 0.010	69.304 ± 0.036	0.744 ± 0.000	14.002 ± 0.082	38.232 ± 0.023
Run 6	3.683 ± 0.064	60.901 ± 0.068	0.575 ± 0.004	10.988 ± 0.036	32.271 ± 0.102
Run 7	5.799 ± 0.057	87.980 ± 0.012	1.882 ± 0.009	12.482 ± 0.045	46.650 ± 0.096
Run 8	4.002 ± 0.089	62.521 ± 0.007	1.429 ± 0.003	10.362 ± 0.019	33.901 ± 0.010

All values are expressed in mg/g extract. nd—not detected. RT—retention time.

**Table 6:** Content in volatile and organic components of E7×3 extract after different storage periods at 40 °C/75% relative humidity (RHu).

Extract	Time at 40 °C at 75% RHu (Days)	Volatile Components	Organics Combustion	Dried Residue
		(% wt vs. Dry Mass)		
E7×3	0	44.4	51.1	2.1
	7	34.4	49.2	1.6
	14	33.5	46.8	2.0
	28	34.9	45.0	3.4

## Data Availability

All data is presented in the articles.

## Supplementary Materials

The following supporting information can be downloaded at: https://www.mdpi.com/article/10.3390/molecules28155795/s1, Figure S1: A-TG and DTG analysis and B-DTA analysis for E7×3 extract prior and after exposure to high humidity atmosphere; Figure S2: HPLC–PDA chromatogram at 323 nm for E7×3 extract initially and after 7, 14 and 28 days of storage in high RHu atmosphere (PC—protocatechuic acid, CfA—caffeic acid, CA—chlorogenic acid, RH—rutin hydrate); Figure S3: antioxidant activity of E7×3 extract with no treatment or with 7–28 days of storage in high RHu atmosphere at 40 °C tested after 10 months of storage.
