# Supplementary material for: Optimisation of Polyphenols Extraction from Wild Bilberry Leaves—Antimicrobial Properties and Stability Studies
Source: Molecules. 2023 Aug 1;28(15):5795. doi: 10.3390/molecules28155795 (PMC10420792; doi:10.3390/molecules28155795)
Supplement: Supplementary file 1 [file molecules-28-05795-s001.zip › molecules-2509288-supplementary.pdf]

Optimisation of Polyphenols Extraction from Wild Bilberry Leaves—Antimicrobial Properties and Stability Studies

Ana-Maria Brezoiu [1], Mihaela Deaconu [1], Raul-Augustin Mitran [2], Ana-Maria Prelipcean [3], Cristian Matei [1] and Daniela Berger [1,*]

[1] Faculty of Chemical Engineering and Biotechnologies, University Politehnica of Bucharest, 1-7 Gheorghe Polizu Street, 011061 Bucharest, Romania; ana_maria.brezoiu@upb.ro (A.-M.B.); mihaela.deaconu@upb.ro (M.D.); cristian.matei@upb.ro (C.M.)
[2] "Ilie Murgulescu" Institute of Physical Chemistry, Romanian Academy, 202 Splaiul Independentei, 060021 Bucharest, Romania; raul.mitran@gmail.com
[3] National Institute of R&D for Biological Sciences, 296 Splaiul Independetei, 060031 Bucharest, Romania; anamaria.prelipcean@incdsb.ro
* Correspondence: daniela.berger@upb.ro

The main effects when heating the extract in the temperature range of 25 °-1000 °C can be seen in Figure S1. The main events are associated with water loss, volatile compounds loss, combustion of organic substances with a solid residue formation (Figure S1A). Figure S1B shows that the combustion of organic substances is highly exothermic.

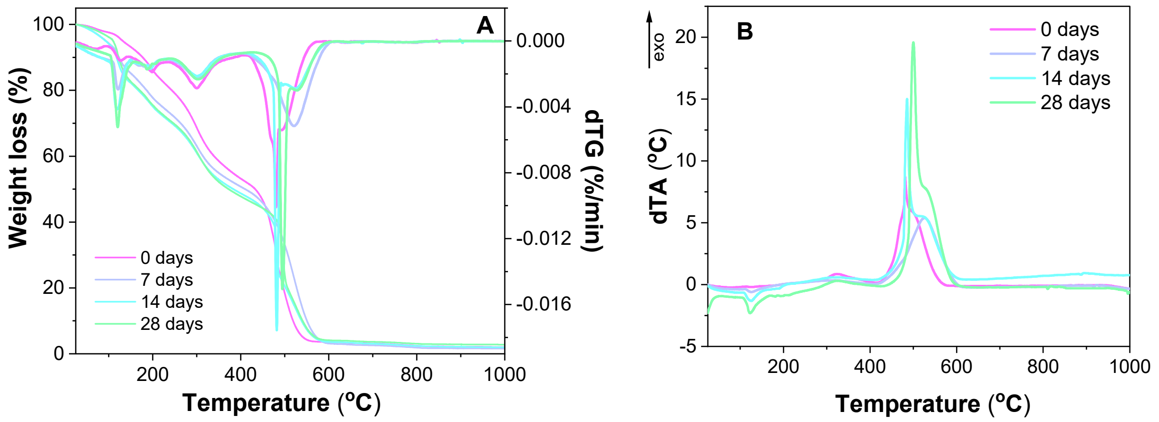

Figure S1. A- TG and DTG analysis and B-DTA analysis for E7x3 extract before and after exposure to high humidity atmosphere.

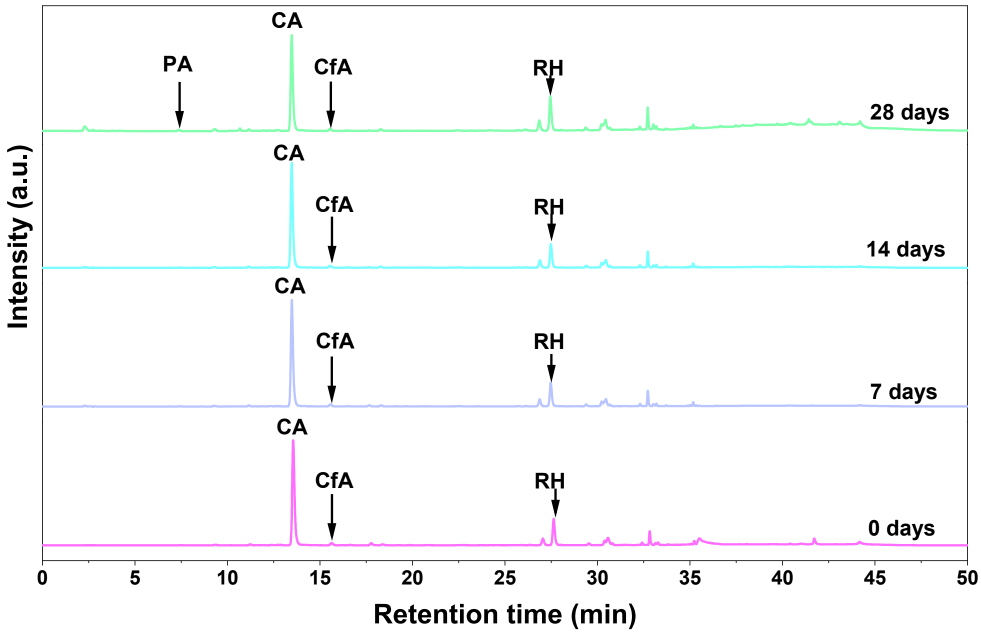

Figure S2. HPLC-PDA chromatogram at 323 nm for E7x3 extract initially and after 7, 14, and 28 days of storage in high RHu atmosphere (PC-protocatechuic acid, CfA-caffeic acid, CA-chlorogenic acid, RH-rutin hydrate).

The assessment of antioxidant activity after 10 months of storage of E7x3 sample showed that the extract stored in dark, at 4 °C, preserved better the radical scavenging properties (609.1±13.5 mg ET/g extract - DPPH assay and 378.8±19.6 mg ET/g extract - ABTS method) compared to the samples exposed at high humidity atmosphere, at 40 °C for 7, 14 and 28 days and then stored in the same conditions, which suffered a significant antioxidant loss (314.2-331.2 mg ET/g extract - DPPH method, and 191.1-238.8 mg ET/g extract - ABTS assay) (Figure S3).

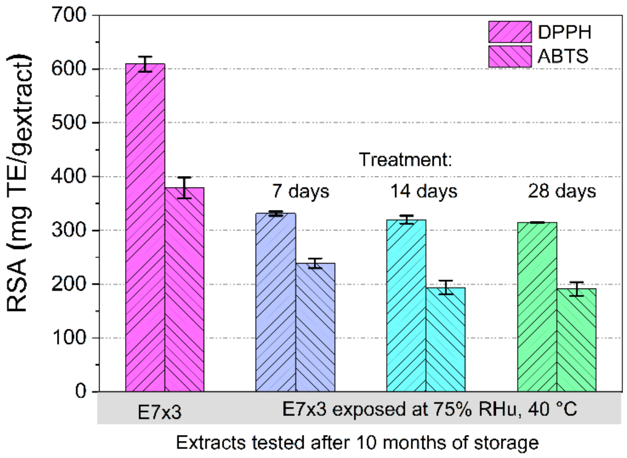

Figure S3. Radical scavenger activity of E7x3 extract with no treatment or with 7, 14, or 28 days of storage in high RHu atmosphere at 40 °C tested after 10 months of storage.
